# Letter to editor regarding “piR-36249 and DHX36 together inhibit testicular cancer cells progression by upregulating OAS2”

**DOI:** 10.1016/j.ncrna.2023.08.007

**Published:** 2023-08-18

**Authors:** Juan Pablo Tosar

**Affiliations:** Functional Genomics Laboratory, Institut Pasteur de Montevideo, Uruguay; Analytical Biochemistry Unit, Nuclear Research Center. School of Science, Universidad de la República, Uruguay

## Abstract

Several reports describing PIWI-interacting RNAs (piRNAs) in human cancer cells or in the bloodstream are affected by the presence of false positives in piRNA databases. A recent report suggested that piR-36249 regulates testicular cancer progression by engaging with DHX36 to regulate OAS2. However, piR-36249 is a tRNA-Cys 5’ half capable of forming intermolecular G-quadruplexes. It is therefore expected that DHX36, a helicase with high affinity for DNA and RNA G-quadruplexes, was pulled down using piR-36249 mimicking probes. The suggestion of using piR-36249 as a therapeutic target for testicular cancer is therefore questionable, due to the consequences that tRNA inhibition could have on healthy cells.

To the Editor,

I have read the manuscript by Wang et al. recently published in *Noncoding RNA Research*, suggesting that the piR-36249/DHX36/OAS2 axis regulates testicular cancer progression [1]. While the paper includes several interesting experiments, I am afraid it is affected by the PIWI-interacting RNA (piRNA) annotation problem that is widespread in the mammalian somatic piRNA literature [2,3].

piR-36249 (DQ598183) is a tRNA-Cys 5’ half. The same genomic region in chromosome 17 that is shown in Wang et al. [1] as the loci producing piR-36249 is annotated in the UCSC Genome Browser as tRNA-Cys, anticodon GCA (see HUGO or RepeatMasker tracks). Consistently, the sequence of piR-36249 is identical to the first 30 nucleotides of tRNA-Cys-GCA-2 based on the genomic tRNA database (http://gtrnadb.ucsc.edu/).

Like tRNA-Cys 5′ halves, piR-36249/DQ598183 starts with 5 consecutive guanines. This 5′ terminal oligoguanine (5′ TOG) motif induces the tetramerization of tRNA-Cys 5′ halves, due to the formation of intermolecular G-quadruplex structures [4,5]. Tetrameric tRNA-Cys 5′ halves regulate translation by interacting with eIF4G [6,7]. The differences observed by Wang et al. in cell viability, proliferation and migration when using piR-36249 mimics vs. control oligonucleotides or inhibitors can therefore be explained by the lack of the 5′ TOG motif in the control sequences. Additionally, while the biotinylated piR-36249 probe used for pull-down experiments contained the 5’ TOG motif, the control probe did not. It is therefore not surprising that DHX36 was experimentally identified as a piR-36249 interactor [1], because DHX36 binds G-quadruplexes with extremely high affinity [8].

In their conclusion, Wang et al. state that their data supports piR-36249 as a novel therapeutic target in testicular cancer [1]. However, targeting a tRNA gene would probably be toxic.

This article [1] illustrates why avoiding mis-classifying noncoding RNA fragments as piRNAs is of paramount importance. While somatic piRNA expression is well documented in arthropods, mollusks, and birds [[9], [10], [11]], piRNA expression in human cancer and the presence of piRNAs in the bloodstream remains to be demonstrated (despite a conundrum of papers suggesting so).

We have introduced the term “miscellaneous piRNAs” (*m*-piRNAs) to contemplate the possibility of certain noncoding RNA fragments being associated with PIWI proteins under certain conditions [3]. However, no direct evidence linking piR-36249 and PIWI proteins was provided by Wang et al. so this piRNA should be considered a wrongly annotated tRNA-derived fragment rather than a canonical piRNA or an *m*-piRNA.

While results in Wang et al. are still valid, the interpretation of these results and the likelihood of using piR-36249 as a therapeutic target should be revisited.

## Declaration of competing interest

The author has no competing interests regarding this submission.
